# 

**Published:** 1906-10-27

**Authors:** 


### BOOKS RECEIVED.

Allman and Son, Limited.

"Food for the Sick." By Mary Truman and Edith Sykes. Third Edition.

Baillière, Tindall, and Cox.

"Questions and Answers on Midwifery for Midwives." By A. B. Calder, M.B.

"Retro-Peritoneal Hernia." By Dr. G. A. Moynihan, M.S. Second Edition.

"Clinical Lectures on Diseases of the Lungs." By J. A. Lindsay, M.D. Second Edition.

"Enlargement of the Prostate." By P. J. Freyer, M.D. Third Edition.

Cassell and Co., Limited.

"Submucous Excision of Deviations and Spurs of the Nasal Septum." By St. Clair Thomson, M.D.

W. B. Clive.

"Matriculation Directory." No. XLIV. September 1906.

Rebman, Limited.

"Minor Surgery and Bandaging." By H. R. Wharton, M.D. Sixth Edition.

"Menstruation and Skin Diseases." By L. D. Bulkley, M.D.

"Skin Diseases and Internal Disorders." By L. D. Bulkley, M.D.

"Diseases of Women." By J. Bland-Sutton, F.R.C.S., and A. E. Giles, M.D. Fifth Edition.

J. Wright and Co.

"Golden Rules of Medical Evidence."

### NOTICE TO CORRESPONDENTS.

**All MSS., Letters, Books for Review, and other matters intended for the Editor should be addressed The Editor, "The Hospital," The Hospital Buildings, 28 & 29 Southampton Street, Strand, W.C.**

**The Editor cannot undertake to return rejected MS., even when accompanied by stamped directed envelope.**

### Notice to Advertisers and Subscribers.

**All Advertisements, Orders for Copies of The Hospital, and Business Communications should be addressed to THE MANAGER (Not to the Editor), The Hospital, 28 & 29 Southampton Street, Strand London, W.C.**

**The Cost of Subscription, post free, is as follows:—Per week, 3½d.; per quarter, 3s. 3d.; per half-year, 6s. 6d.; per annum, 13s. Foreign and Colonial:—Per annum, 19s. 6d., payable, in all cases, in advance.**

### MUSCLES AND NERVES

**An Atlas of the Superficial Muscles and the Principal Motor Nerves of the Human Body for the use of Students of Anatomy and Nurses.**

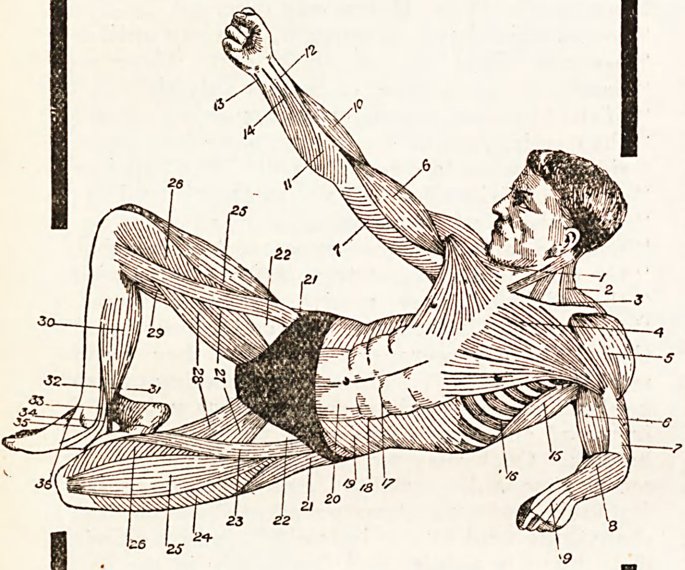

By LOUIS B RAWLING, F.R.C.S.,
*Assistant Surgeon and Senior Demonstrator of Anatomy, St. Bartholomew's Hospital.*

**Price 3/6 net, 3/9 post free.**

This Atlas contains a series of photographs of a well-developed male in such positions calculated to show off the superficial muscles to the best advantage. Opposite to each figure is a plan which not only illustrates the arrangement and direction of the muscles called into action, but also indicates the situation of the more important motor nerves.

The work cannot fail to be of the greatest practical utility to all Nurses and those who desire to gain a thorough insight into the superficial muscular formation of the human body, and obtain a general idea as to the position of the nerves which supply those muscles.

*Of all Booksellers, and of*

THE SCIENTIFIC PRESS, Limited,
28 & 29 SOUTHAMPTON STREET, STRAND,
LONDON, W.C.

### 1/6 NURSING HANDBOOKS.

By Post, 1/8

### SURGICAL INSTRUMENTS AND APPLIANCES USED IN OPERATIONS.

**An Illustrated and Classified List, with Explanatory Notes.**

By Harold Burrows, M.B.Lond., B.S., F.R.C.S.
New Edition, Revised and Enlarged.

### ART OF FEEDING THE INVALID.

By a Medical Practitioner and a Lady Professor of Cookery.
New and Popular Edition, with Special Chapter on Diet for Consumptives.

### THE CARE OF CHILDREN.

**Practical Hints for Mothers and Nurses at Home and Abroad.**

By Robt. J. Blackham, D.P.H.Lond., C.B.
Revised and Enlarged Edition.

### LECTURES ON HOME NURSING FOR THE POOR.

By a District Nurse.
Illustrated.

### OUTLINES OF ROUTINE IN DISTRICT NURSING.

By M. Loane, Superintendent of District Nurses, Portsmouth.
1/9 post free.

### OUTLINES OF INSANITY.

**A Popular Treatise on the Salient Features of Insanity.**

By Francis H. Walmsley, M.D.

Popular Edition.

### NURSING OF TROPICAL FEVERS.

By S. F. Pollard, late Sister, Army Nursing Reserve.
The Work is based upon the Personal Experience of the Author Abroad.

*Complete Catalogue post free on application.*

THE SCIENTIFIC PRESS, Ltd.,
Nursing Publishers and Booksellers,
28 & 29 SOUTHAMPTON STREET,
STRAND, LONDON, W.C.

